# Applications of Percolation Theory to Prevent the Propagation of Phytopathogens and Pests on Plantations

**DOI:** 10.3390/e27040386

**Published:** 2025-04-05

**Authors:** J. Alonso Tlali, J. R. Alvarado García, B. Cardenas Castro, A. Fernández Téllez, E. G. García Prieto, J. F. López-Olguín, Y. Martínez Laguna, J. E. Ramírez, D. Rosales Herrera, J. D. Silva Montiel

**Affiliations:** 1 Facultad de Ciencias Físico Matemáticas, Benemérita Universidad Autónoma de Puebla, Apartado Postal 165, Puebla 72000, Puebla, Mexico; 2 Facultad de Ciencias Biológicas, Benemérita Universidad Autónoma de Puebla, Apartado Postal 165, Puebla 72000, Puebla, Mexico; 3 Centro de Agroecología, Instituto de Ciencias, Benemérita Universidad Autónoma de Puebla, Apartado Postal 165, Puebla 72000, Puebla, Mexico; 4 Herbario y Jardín Botánico, Vicerrectoría de Investigación y Estudios de Posgrado, Benemérita Universidad Autónoma de Puebla, Apartado Postal 165, Puebla 72000, Puebla, Mexico; 5 Vicerrectoría de Investigación y Estudios de Posgrado, Benemérita Universidad Autónoma de Puebla, Apartado Postal 165, Puebla 72000, Puebla, Mexico

**Keywords:** percolation threshold, complex network, agroecology, intercropping plantation, *Phytophthora*

## Abstract

One of the most important problems in agroecology is designing eco-friendly strategies to minimize the propagation of phytopathogens and pests. In this paper, we explore some strategies based on the modification of the plantation configuration together with percolation theory to prevent the propagation of phytopathogens and pests that move over nearest neighbor plants, such as the case of *Phytophthora* zoospores or pest mites. The percolation threshold is determined for well-mixed and intercropping plantations modeled in nearest neighbor square lattices. Our main result is that the best agroecology strategy consists of designing polyculture plantations to raise the net production yield.

## 1. Introduction

Percolation theory is a branch of statistical physics based on geometric and probabilistic arguments to study the flow of a fluid through a porous medium, following the general ideas of the study of critical phenomena. It was formally introduced in 1957 by Broadbent and Hammersley when they discovered that the amount and connectivity of charcoal pores determined its effectiveness in filtering toxic gases [1]. This discovery, along with similar results at the time, led scientists to conclude that a new type of critical phenomenon was involved wherein the passing of a fluid through a random medium is determined by the medium’s structure [2,3,4].

The minimal description that percolation theory uses to model transport phenomena is through a collection of adjacent sites (or nodes) independently occupied (or linked) with probability *p*. The adjacent occupied sites form clusters whose sizes increase with the occupation probability *p*. Notice that for the case p→0, the medium has small isolated clusters that hinder the flow. Otherwise, when p→1, there is probably a path of adjacent occupied sites that facilitates the flow from point A to point B [1,3]. In this context, the clusters’ size variations as a function of the system parameters are of interest [3]. The fundamental problem that percolation theory solves is determining the minimum occupation probability $p_{c}$ at which a spanning cluster emerges and extends across the entire system. This critical probability, also referred to as the percolation threshold, marks the onset of a phase transition where the nonpercolating system changes to a percolating state [3]. Here, the main observables exhibit power law behaviors around the percolation threshold characterized by critical exponents, as expected in phase transition theory [5].

Since percolation theory was formally introduced in the literature, it has been applied in a wide range of research fields by describing the connection properties among the elements of a complex system and their macroscopic effects [6,7]. Although it might seem straightforward, the implementation of percolation theory requires an adequate description of the system, the interactions between its constituents, and their interpretation within the framework of percolation theory [8,9]. For instance, in high-energy physics, the two-dimensional continuum percolation theory with fully overlapping disks has been applied to investigate the quark gluon plasma formation [10], a state of matter believed to exist within the initial milliseconds after the Big Bang [11]. In this model, the formation of clusters arises from the overlap of the disks, which represent the color interactions between the colliding partons. Consequently, the formation of the quark gluon plasma is tied to the emergence of the spanning cluster of color strings [10,12,13,14,15]. Another example is presented in Ref. [16], where the authors modeled the municipalities of a state as a random network linked by the highway system to examine the spread of COVID-19. Here, the percolation threshold was associated with the percentage of municipalities that needed to limit their mobility to prevent a significant outbreak (spanning cluster).

This paper is motivated by the spread of diseases in plantations caused by phytopathogens or pests with self-mobility, which is addressed under the percolation theory framework. In particular, *Phytophthora* zoospores (from the Greek *phyto*, meaning “plant”, and *phthora*, “destroyer”) can spread directly to nearest neighbor plants by chemotactically moving over soil moisture or water films [17,18]. Once *Phytophthora* infects a plant, it rots the roots, causing fruit loss and the plant’s death [19]. Many species of *Phytophthora* can persist as saprophytes for extended periods if the environmental conditions are not appropriate but become parasitic in the presence of susceptible hosts [20,21]. Consequently, *Phytophthora* causes damage not only to the economy but also to the environmental sector. Another important agricultural pest that spreads to neighboring plants through self-mobility is the red spider mite (*Tetranycus urticae*), which moves across the surface of plants and travels along paths of touching leaves [22]. Similarly to *Phytophthora*, the red spider mites affect crops of high agroecological interest, such as tomatoes, potatoes, beans, and corn, among other vegetables [22]. This pest feeds by sucking the sap from the leaves, causing significant foliar damage that impedes photosynthesis, which withers and kills the plant [22]. One of the main techniques for controlling phytopathogens and pests is through chemical control, which in the case of the red spider mites has shown a certain degree of success. However, the main consequences of this management are the contamination of fruits as well as the development of resistance to chemical products. In contrast, there are no effective chemical treatments in the case of *Phytophthora* [23,24,25]. Therefore, studying the geometry of plantations within the percolation theory scheme becomes necessary to develop and evaluate agroecological strategies to prevent the spread of diseases and pests.

In this paper, we model plantations as square lattices where each cell is planted. The plant–phytopathogen interaction is incorporated by introducing the plant’s susceptibility, which measures the probability of a plant becoming ill after exposure to the phytopathogen. In this context, occupied sites correspond to susceptible plants. Since *Phytophthora* can move through their flagella, it can spread from plant to plant, resembling a percolation problem on nearest neighbor square lattices. We define the percentage of cells with the phytopathogen at the beginning of the propagation process to include the information on the presence of *Phytophthora* on the soil’s plantation. This parameter is relevant because the propagation starts from them. In some instances, the *Phytophthora* zoospores’ mobility affects the plantation’s connectivity by connecting susceptible plants beyond the nearest neighbor vicinity. This effect accelerates the formation of larger clusters of ill or dead plants. One agroecological strategy to avoid the zoospores’ spread is changing the plantation’s configuration by introducing a second, less susceptible plant type. In particular, we study the connectivity of the following planting configuration: well-mixed plantation and intercropping plantations by intercalating columns or diagonals. The latter holds significant interest because they resemble “milpa” systems, which are ancient polyculture systems widely used in Mexico. In this context, we found critical curves that are understood as the percolation threshold that prevents the spread of the phytopathogen. Therefore, the most resistant plant serves as a natural barrier protecting the susceptible ones. Interestingly, we also found that despite having abundant inoculated cells at the beginning of the propagation process, there are pairs of plants that allow farmers to increase the net production yield. The application of the results presented in this work is illustrated by comparing the critical curves with pairs of experimentally determined susceptibilities for three types of chili peppers with high commercial value.

The rest of this manuscript is organized as follows. In Section 2, we introduce the percolation model on monoculture plantations and define the main parameters of our model. In Section 3, we describe the effect of the phytopathogen self-mobility on the plantations’ connectivity. In Section 4, we discuss the computational implementation to determine the percolation thresholds for well-mixed and intercropped planting configurations as a function of the number of inoculated cells at the beginning of the propagation process. The results of the percolation threshold are reported in Section 5. Finally, in Section 6, we present the conclusions of this work.

## 2. Monoculture Plantations

**Figure 1:**
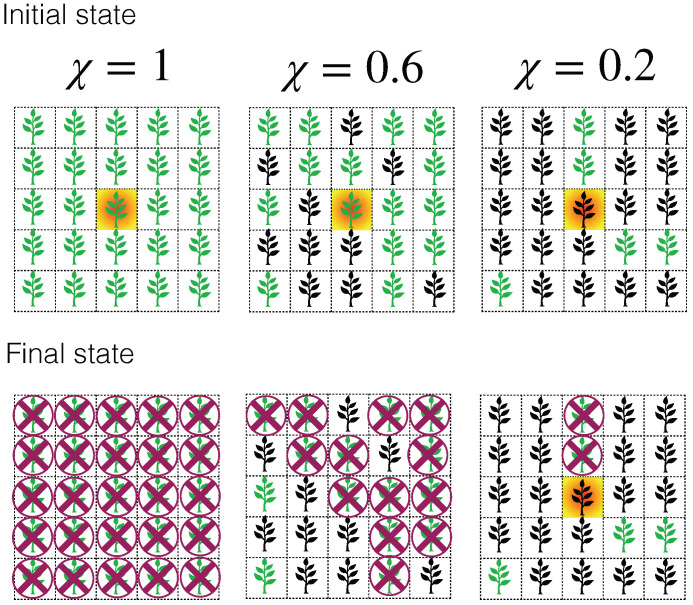
Sketch illustrating the propagation of *Phytophthora* zoospores on a plantation with varying susceptibility values: 1.0 (left), 0.6 (middle), and 0.2 (right). In the initial state, there is only one inoculated cell (shaded cell) that propagates among susceptible plants (green plants), while the resistant plants (black plants) remain unaffected and play the role of barriers protecting susceptible plants. At the end of the propagation, the final state shows extensive damage (crossed cells) for plantations with susceptibilities above the percolation threshold $\chi_{c}$ = 0.59274621(13).

Monoculture plantations are one of the most extensive agroecological practices worldwide [26]. They involve growing only one crop type at a time on a specific field, usually arranged in regular structures like square or triangular (quincunx planting) lattices. We use this fact to model plantations as square lattices with spacing of the maximum length that the *Phytophthora* zoospores can travel before entering a dormancy state [27]. We incorporate the information on the plant–phytopathogen interaction by introducing the susceptibility (χ) as the probability of a plant becoming ill after exposure to the phytopathogen [27]. The susceptibility can be determined either experimentally or in situ by observing the survival rate of plants that are effectively exposed to the phytopathogen [27]. It is found that some individuals deploy defense mechanisms against the zoospores’ infestation, but there are no quantitative or qualitative methods to determine which seed will grow as a resistant or a susceptible plant [27,28]. Therefore, we consider that susceptible (or resistant) individuals are uniformly distributed along the plantation. Under these conditions, the disease only propagates on susceptible adjacent plants, similar to a percolation process occurring in the nearest neighbor square lattice, as we depict in Figure 1. Thus, the susceptibility takes the role of the occupation probability for fully sown plantations, with a critical susceptibility $\chi_{c}$ = 0.59274621(13) [29,30]. In Figure 1, we sketch the propagation of *Phytophthora* zoospores on a plantation with different susceptibility values.

It is worth mentioning that resistant plants act as barriers, protecting susceptible ones. Therefore, it is crucial to examine, through soil tests, the microbiota in the plantation’s soil and their interactions with the specific plant variety of interest. In Table 1, we show the experimental determination of the susceptibility of different chili pepper varieties (of great commercial value in Mexico) exposed to different *Phytophthora capsici* strains (for further details, see Ref. [27]).

**Figure 2:**
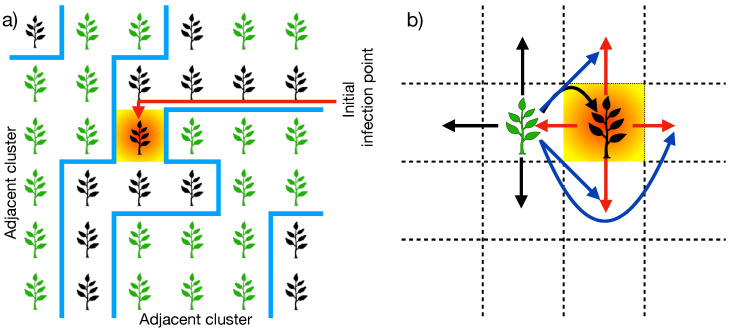
(**a**) Sketch depicting a scenario where an inoculated cell at the beginning of the propagation process (shaded cell) is positioned within a resistant plant (black plant) and is adjacent to two disjoint clusters of susceptible plants (green plants). (**b**) Diagrammatic representation of how the zoospores’ mobility extends the nearest neighbor vicinity (black and red arrows) by connecting susceptible plants in the next-to and next-next-to nearest neighborhoods (blue arrows). This occurs when an inoculated cell also contains a resistant plant adjacent to clusters of susceptible plants.

Another relevant parameter for the model is the percentage of inoculated cells at the beginning of the spreading process (*I*), from which the disease propagation starts. For simplicity, we consider that the inoculated cells are initially uniformly distributed on the plantation. Here, we must note that the mobility of the zoospores may affect plantation connectivity in situations where the cells are inoculated and contain resistant plants [31]. One cell satisfying these conditions can connect two disjoint finite clusters far from the neighborhood definition, as depicted in Figure 2a. This situation modifies the nearest neighbor definition for the cells under the conditions mentioned earlier (see Figure 2b), which promotes the emergence of the spanning cluster at susceptibilities below the percolation threshold. In Section 3, we further discuss the effects of the percentage of inoculated cells at the beginning of the spreading process in the critical susceptibility.

## 3. Zoospores’ Mobility Affects the Plantation Connectivity

**Figure 3:**
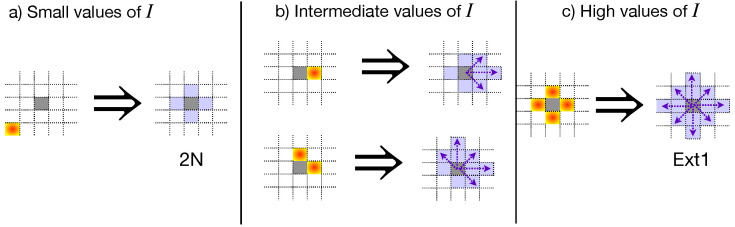
Modification of the neighborhood definition as the percentage of inoculated cells at the beginning of the propagation process increases. Purple-shaded cells indicate the possible neighbors of a susceptible plant (gray-shaded cells) under the presence of inoculated cells (gradient-filled). (**a**) At low *I* values, the inoculated cells have a low probability of satisfying the conditions required to form bridges between susceptible plants far away from the nearest neighborhood (purple arrows). (**b**) However, as *I* increases, the inoculated cells begin to connect susceptible plants to the next-to-nearest vicinity. (**c**) For high values of *I*, the mobility of zoospores resembles a square lattice with an extended neighborhood beyond the next-to-nearest neighbors. In all cases, the percolation threshold modification is a consequence of the collective phenomena of the zoospores’ mobility, affecting the plantation connectivity.

The spread of phytopathogens, such as the *Phytophthora* zoospores, has been studied as a percolation problem [27,31,32,33], where the percolation threshold depends on the plantation’s geometry and the percentage of inoculated cells at the beginning of the propagation process. These inoculated cells are relevant because they initiate the propagation process. In particular, if the inoculated cells are also empty or contain a resistant plant, they act as bridges connecting susceptible plants beyond the neighborhood definition. Figure 3 illustrates this phenomenon for low, intermediate, and high values of *I*. Notice that as *I* increases, the inoculated cells start to connect plants far away from the nearest neighbor vicinity. Thus, the percolation threshold decreases as a response to the collective phenomena produced by zoospores’ mobility [31].

To model the illness spreading in monoculture systems, the plantation is represented as a regular lattice with two independent occupancy states: inoculation and the presence of susceptible plants. Initially, inoculated cells are distributed uniformly and independently of their neighbors. Subsequently, following the Newman–Ziff algorithm [29], susceptible plants are incorporated one by one, performing the clustering if the adjacent sites are occupied with a susceptible plant or inoculated. Despite its simplicity, the presence of inoculated cells significantly influences the cluster formation and the percolation threshold. The simulation ends when a spanning cluster of susceptible or diseased plants is observed, and the percolation threshold is estimated from the data generated. In this sense, propagation only connects susceptible plants. Here, the susceptibility plays the role of the occupation probability in traditional percolation theory. Thus, the percolation threshold is associated with the critical susceptibility $\chi_{c}$, meaning that the plantation must be sown with plants whose susceptibility is lower than $\chi_{c}$ to avoid outbreaks [33].

It was observed that $\chi_{c}$ decreases with an increasing number of extended sites, which is controlled by the probability parameter *I* [32,33]. However, there is a minimum value of susceptibility that allows the entire plantation to be sown even with complete inoculation. Considering this result in plantation management is key to preventing outbreaks in subsequent agricultural cycles, especially because *Phytophthora* can survive in adverse environmental conditions and its propagation can increase when those conditions vary favorably.

Notice that the agroecological model discussed above introduces a redefinition of the neighborhood for traditional nearest-neighbor square lattices. In particular, the 2N plantations refer to planting configurations with the nearest neighborhoods. In the presence of inoculated cells, 2N plantation resembles composite square lattices, and the critical susceptibility is bounded as $p_{c,2N+3N}\leq \chi_{2N}\leq p_{c,2N+Ext1}$, where $p_{c,reg+ext}$ is the percolation threshold for the composite systems with regular (*reg*) and extended (*ext*) neighborhoods. Here, 3N and Ext1 denote the next-to-nearest and next-to-next-nearest neighborhoods, respectively [31,32,33]. Furthermore, the critical susceptibility can be accurately described by the following linear combination:

(1) $$\chi_{2N}=\left(1-I\right)p_{c,2N+3N}+Ip_{c,2N+Ext1},$$

which, for low *I* values, behaves as the exponential decay

(2) $$\chi_{2N}∼\exp\left(-I/\lambda^{\prime }\right),$$

where $\lambda^{\prime }=\lambda_{2N+3N}/\left(1-\frac{p_{c,3N}}{p_{c,2N}}\right)$. Here, $\lambda_{2N+3N}$ is a geometrical factor that modulates the exponential decay of the 2N+3N composite system, which can be expressed in terms of the gyration radius of the regular ($R_{reg}$) and extended ($R_{ext}$) neighborhoods [33].

**Figure 4:**
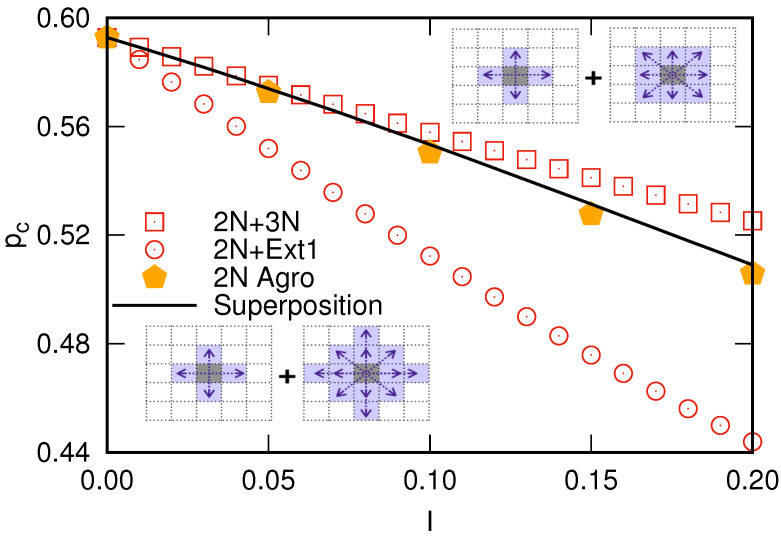
Percolation threshold of composite square lattices 2N+3N (squares), 2N+Ext1 (circles), and the monoculture plantations (pentagons) with increasing the number of inoculated cells at the beginning of the propagation process. The solid line is the superposition of the percolation threshold for composite square lattices that perfectly matches the agroecological model.

Figure 4 presents the percolation thresholds for the 2N+3N and 2N+Ext1 composite systems as a function of *I*, showing that the critical susceptibility for 2N plantation lies between these values, satisfying the boundaries discussed above. The superposition (1) captures the transition of the 2N plantations to composite systems as the percentage of inoculated cells at the beginning of the propagation process increases. Therefore, the zoospores’ mobility makes the propagation process complex, requiring a more elaborated description, which includes hybrid models of composite systems rather than the traditional percolation. It is worth mentioning that the 2N plantations start out being totally like the traditional nearest neighbor square lattices (in the limit I→0), transitioning to the 2N+Ext1 case up to the limit I=1 but passing for the 2N+3N composite systems for intermediate *I* values [33], as illustrated in Figure 3.

To close this section, we must emphasize that the appropriate composite percolation system can adequately model the propagation of phytopathogens and pests moving through adjacent plants. This is because introducing the inoculated cells at the beginning of the propagation process affects the plantation’s connectivity, taking an important role in the percolation threshold. The study of the critical susceptibility for these systems provides insights into how the plantation geometry and inoculation level influence the propagation in 2N plantations, highlighting the importance of planting configuration and the acknowledgment of the plant–phytopathogen interaction in the control and management of the disease to mitigate outbreaks, particularly in the presence of resilient phytopathogens like *Phytophthora*.

## 4. Agroecological Strategies, Simulation, and Data Analysis

In this section, we discuss agroecological strategies focused on plantation configuration to prevent the spread of phytopathogens and pests that can move from one plant to adjacent ones. We also detail their computational implementation and data analysis.

### 4.1. Agroecological Strategies

**Figure 5:**
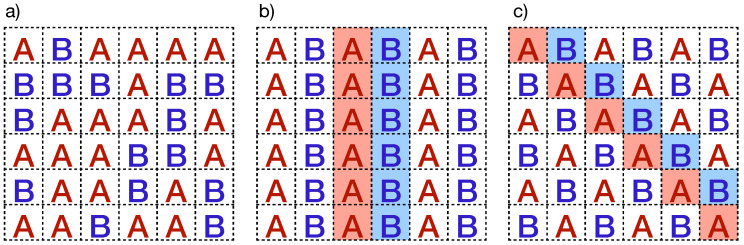
Planting configurations with two plant types presented in this work: (**a**) well-mixed configuration and intercropping plantations where A and B plant types are sown by intercalating (**b**) columns and (**c**) diagonals.

Polyculture systems are straightforward strategies for planting configurations involving the cultivation of two or more plant varieties in the same field. In the literature, many benefits of polyculture systems are reported, including nutrient fixation in the soil, enhancement of microbiota, promotion of biodiversity, and crop protection, among others. Thus, we adopt the strategy of planting two varieties of plants (*A* and *B*), one more resistant than the other, to diminish the connectivity of susceptible plants in the plantation. We explore three configurations: (i) well-mixed plantation at different proportions, (ii) intercropping by intercalating columns, and (iii) intercropping by intercalating diagonals (chess-like plantation). Figure 5 depicts these planting configurations. Additionally, we only consider fully planted fields, meaning all sites in the lattices are occupied by plants.

In the well-mixed plantations, plant varieties *A* and *B*, with susceptibilities $\chi_{A}$ and $\chi_{B}$, are randomly planted (see Figure 5a). In this particular case, we also consider that a fraction Mix of the total cells is sown with the *A*-plant type. Therefore, the remaining sites are occupied with the *B*-plant type. In some instances, there are theoretical predictions where the well-mixed plantations can increase the total yield production, as discussed in Ref. [27]. However, the control and management of well-mixed plantations may not be suitable for arbitrary plants because different varieties of plants could need specific nutrients and care. In such cases, intercropping systems are alternative planting configurations designed with specific structural and periodic patterns. In particular, we study arrays wherein the field is planted by interspersing complete columns sown by *A*- and *B*-plant types, as depicted in Figure 5b. Another intercropping option is the chess-like plantation, arranged by intercalating diagonals instead of columns, as depicted in Figure 5c. Remarkably, “milpa” systems in Mexico represent an ancient agrobiodiverse practice of intercropping plantation [34]. In these polycropping systems, maize, squash, beans, or other legumes are seeded in an intercropped configuration. Notice that the plantation connectivity is modified in all planting configurations. In fact, in chess-like plantations, if the $\chi_{B}<\chi_{A}$, the transmission of the zoospores is limited by the *B*-type plants, which act like physical barriers that protect the susceptible plants. In Section 4.2, we provide a detailed discussion of our computational implementation used to estimate the percolation threshold for the three planting configurations described above.

### 4.2. Simulation and Data Analysis

We model the plantations as square lattices, selecting the spacing as the maximum distance the zoospores can travel before starving or entering a dormant state. Given these considerations, disease propagation occurs from infected cells to neighboring susceptible plants, similar to a percolating problem on a nearest neighbor square lattice. In all three planting setups (see Figure 5), we simulate sowing two different plant types, *A* and *B*, with susceptibilities $\chi_{A}$ and $\chi_{B}$, respectively. For the simulation, we employ the Newman–Ziff algorithm to estimate the percolation threshold as follows [29,35]. The square lattice of size $L^{2}$ is linearized using the mapping M=iL+j, where i,j=0,1,…,L−1 correspond to the cell entries in a matrix representation. This procedure enables us to compile a list of labels that are helpful in the clustering process, which employs the Union–Find algorithm. In all cases, we estimate the critical susceptibility of the *B*-type plant ($\chi_{B}$) by fixing in the simulation the values of the *A*-type plant susceptibility ($\chi_{A}$), the percentage of inoculated cells at the beginning of the propagation process (*I*), or the proportion between the plant varieties (Mix), according to the planting configuration.

In the simulation, we first explore the sites corresponding to the *A*-type plants. For the well-mixed plantation, the number of *A*-type plants ($n_{A}$) is randomly chosen from the binomial distribution $B(L^{2},\chi_{A})$. Conversely, there are $L^{2}/2$ sites assigned to each plant type for the intercropping planting configurations, which are allocated at M=iL+2j, and

(3) $$M=\left\{\begin{array}{cc} iL+2j & \mathrm{if}\;i\;\mathrm{is}\;\mathrm{even}\\ iL+2j+1 & \mathrm{otherwise} \end{array}\right.,$$

for intercropping planting configurations with alternate columns and diagonals, respectively. In the labeling, *i* = 0, ..., L−1 and *j* = 0, ..., L/2−1 in both cases.

We randomly add the *A*-type plants for the well-mixed configuration by sorting the label list in random order, but for the intercropping plantations, they are systematically added by checking all the labels assigned to the *A*-type plants. In each step, the susceptibility state of the plants is decided by generating a uniformly distributed random number ($r_{\chi_{A}}$) in the range [0,1] and comparing it with $\chi_{A}$. Thus, the plant is susceptible if $r_{\chi_{A}}<\chi_{A}$. Otherwise, the plant is resistant. Then, if the added plant is susceptible, we perform the clustering process by checking the occupancy and inoculation state of the adjacent cells, applying the function *Union* if the neighbor site is occupied with a susceptible plant or inoculated.

After allocating the *A*-type plants, we add the remaining labels, which are assigned to the *B*-type plants, one at a time. Similarly to the *A*-type plants allocation case, for each added site, we check the occupation and inoculation state of the adjacent sites, connecting and merging clusters accordingly. This process continues until the spanning cluster emerges, which is detected by inspecting the labels of the left and right borders. At this point, we store the critical number $n_{Bc}$ of *B*-type plants necessary to the spanning cluster emergence. Thus, we estimate the probability $f_{n}$ of the spanning cluster emergence after adding exactly *n* sites as the ratio of the frequency of observing *n* to the total number of simulations. Therefore, the cumulative distribution

(4) $$F_{n}=\sum_{k=0}^{n}f_{k}$$

is the probability of observing the spanning cluster emergence after adding at most *n* sites. Following the Newman–Ziff simulation scheme [29], we convolute the cumulative distribution (4) with the fluctuations of the number of occupied sites at a fixed occupation probability *p*, given by the binomial probability mass function

(5) B(n,Nmax,χB)=NmaxnχBn(1−χB)Nmax−n,

where

(6) $$N_{\max}=\left\{\begin{array}{cc} \left\lfloor \left(1-Mix\right)L^{2}\right\rfloor & \mathrm{for}\;\mathrm{well-mixed}\;\mathrm{plantations}\\ \left\lfloor L^{2}/2\right\rfloor & \mathrm{for}\;\mathrm{intercropping}\;\mathrm{plantations} \end{array}\right.,$$

The percolation probability is estimated as follows [29,35]:

(7) $$P_{L}\left(\chi_{B}\right)=\sum_{n=0}^{N_{\max}}F_{n}B\left(n,N_{\max},\chi_{B}\right).$$

The subindex *L* in Equation (7) denotes the dependence of the percolation probability on the system size. It is well-known that the percolation probability exhibits a sigmoid behavior as a function of the occupation probability [36]. Moreover, the percolation threshold for finite systems is usually estimated as the occupation probability value satisfying $P(\chi_{BcL})=1/2$. Therefore, we fit the function [31]

(8) $$\pi_{L}\left(\chi_{B}\right)=\frac{1}{2}\left(1+\tanh\left(\frac{\chi_{B}-\chi_{BcL}}{\Delta_{L}}\right)\right),$$

to the percolation probability data estimated using Equation (7). In Equation (8), $\chi_{BcL}$ and $\Delta_{L}$ are the estimation of the percolation threshold and the transition width [31,36], respectively. Both $\chi_{BcL}$ and $\Delta_{L}$ exhibit explicit finite size effects that are quantified by the following power laws:

(9) $$\begin{array}{cc} \Delta_{L} & \propto L^{-1/\nu } \end{array}$$

(10) $$\begin{array}{cc} \chi_{Bc}-\chi_{BcL} & \propto L^{-a}, \end{array}$$

where ν is the critical exponent associated with the correlation length, $\chi_{Bc}$ is the estimation of the critical susceptibility of the *B*-type plants in the thermodynamic limit (L→∞), and *a* is an exponent related to the finite size effects of Equation (10). In Section 5, we report our results of the critical curves as a function of the susceptibility $\chi_{A}$ and the percentage of inoculated cells at the beginning of the propagation process for the well-mixed and intercropping planting configurations.

## 5. Results

We run simulations to determine the critical susceptibility of the *B*-type plants when they are sown together with *A*-type plants under different values of the percentage of inoculated cells at the beginning of the propagation process. We consider the values *I* = 0.00, 0.01, 0.05, and 0.10 for each simulated case. Here, the limit I→0.00 means that there is only one inoculated cell in the plantation at the beginning of the propagation process. In the specific case where $\chi_{A}=\chi_{B}$, the plantation connectivity resembles monoculture systems (see Section 2 and Section 3), even when the illness manifests at different times for different plant varieties. For the well-mixed plantations, we establish the percentage of cells sown with *A*-type plants at values of Mix = 0.5, 0.4, and 0.3. Lower Mix values indicate that the plantation is moving towards monoculture systems, requiring more resistant plants to prevent disease dissemination.

We run 10^6^ simulations for each system with fixed values $\chi_{A}$, *I*, and Mix, correspondingly. We also simulate systems with the side values *L* = 128, 192, 256, 384, and 512 to determine the finite size effects on the determination of $\chi_{Bc}$ (total simulations $∼2.5\times 10^{9}$). In all cases, we found 1/ν≈0.75, which is consistent with the universal value reported for the critical exponent associated with the correlation length for two-dimensional percolation systems [37,38]. In contrast, the exponent *a* in Equation (10) takes values ranging from 0.75 to 3, showing clear evidence of the dependence of the finite size effects on the planting configuration. This is because the finite size effects are computed from the addition of the *B*-type plants, which may affect the cluster growing process.

**Figure 6:**
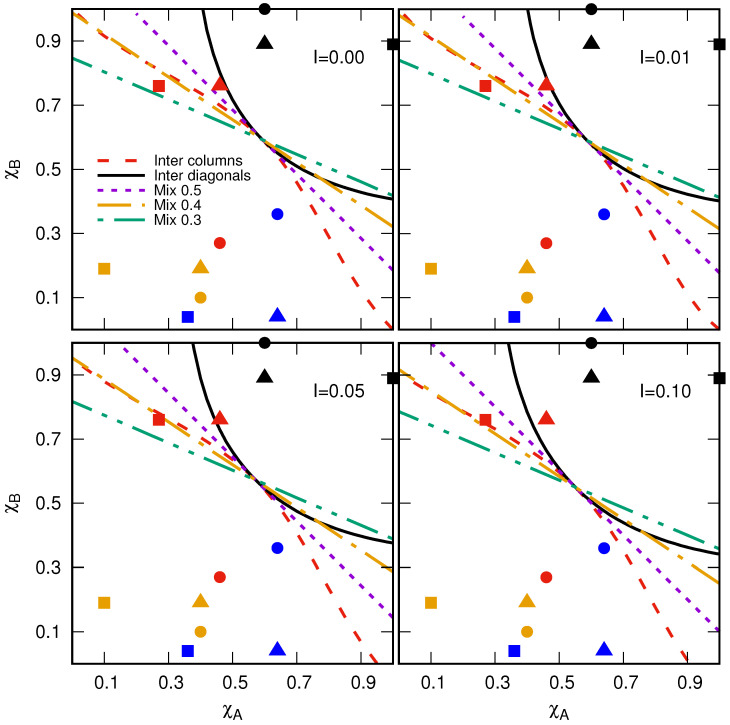
Phase diagrams as a function of *I* for the planting configurations: intercalating columns (dashed line) or diagonals (solid line) and well-mixed with Mix = 0.5 (dotted line), 0.4 (dash-dotted line), and 0.3 (dot-dot-dashed line). The interpretation of the critical curves (lines) is as follows: for a particular pair of plants, if their susceptibilities point is below the critical curve, the propagation process is controlled by occurring on a finite cluster. Otherwise, the disease will invade a large portion of the plantation. Figures correspond to pair combinations of different chili susceptibilities: serrano–árbol (circles), serrano–poblano (triangles), and árbol–poblano (squares) exposed to different isolates of *Phytophthora capsici*: PcV01 (black), PcV51 (red), PcV77 (blue), and PcV90 (yellow) (see Table 1).

In Figure 6, we present our estimation of the critical susceptibilities $\chi_{Bc}$ in the thermodynamic limit under the conditions mentioned above. Notice that in the plane $\chi_{A}-\chi_{B}$, the critical susceptibilities correspond to critical curves separating the region [0,1]×[0,1] in two phases: propagation and nonpropagation of the disease. For an arbitrary pair of plants with susceptibilities $\chi_{A}$ and $\chi_{B}$, the disease propagation will occur on a small part of the plantation if the point $\left(\chi_{A},\chi_{B}\right)$ is below the critical curve, meaning that the propagation process is controlled. Otherwise, the disease will invade a large portion of the plantation, substantially reducing the total yield. All planting configurations respond in the same way to increments of the initial inoculated cells, raising the area where the disease propagation is guaranteed (see Figure 6). This is because an increase in *I* promotes the occurrence of sites with extended neighborhoods, as is discussed in Section 3. In particular, in the limit I→0, the critical curve for the chess-like intercropping plantation matches with the results reported for sublattice-selective percolation on bipartite square lattices [39,40].

Note that the critical curve for the well-mixed planting configurations shows a linear response, as shown in Figure 6. This can be explained in the following way. In a well-mixed plantation with given $\chi_{A}$, $\chi_{B}$, and Mix, the average number of cells available for the disease spreading is $N_{\mathrm{dis}}=\langle N_{A}\rangle +\langle N_{B}\rangle$, where $\langle N_{A}\rangle =NMix\chi_{A}$ and $\langle N_{B}\rangle =N\left(1-Mix\right)\chi_{B}$ are the average number of cells with *A*- and *B*-type plants, respectively, and *N* is the total number of cells in the plantation. We recall that the initial inoculated cells can only act as a bridge connecting sites far from the nearest neighbor if they occupy a site with a resistant plant. Thus, for low *I* values, the average number of sites satisfying this condition is $(N-N_{\mathrm{dis}})I$. Under these conditions, this system corresponds to a percolation process occurring on a square lattice with an effective occupation probability given by $p_{\mathrm{eff}}=I+\left(1-I\right)\left[Mix\chi_{A}+\left(1-Mix\right)\chi_{B}\right]$. Writing down in favor of $\chi_{B}$ and taking $p_{\mathrm{eff}}=p_{c}$, the latter equation can be expressed as follows:

(11) $$\chi_{Bc}=\frac{1}{1-Mix}\left(\frac{p_{c}-I}{1-I}-Mix\chi_{A}\right).$$

Notice that Equation (11) linearly relates the critical susceptibility $\chi_{Bc}$ with $\chi_{A}$ where the slope depends only on the value of Mix. We recall that Equation (11) was derived for fully sowed well-mixed plantations and low values of *I*. Moreover, it is expected that cluster structures and their scaling properties belong to the same universality class of random percolation since the well-mixed plantation resembles a random square lattice with an occupation probability $p_{\mathrm{eff}}$. Conversely, this fact may not hold for structured plantations due to the change in the plantation connectivity when the plant susceptibilities differ.

## 6. Conclusions

In this work, we modeled the propagation of phytopathogens and pests on a plantation as a percolation system. In particular, we discussed the case of *Phytophthora* zoospores, which chemotactically move toward plants by employing their flagella. For simplicity, we adopted square lattice plantations with spacing equal to the maximum distance that zoospores can travel before starving or entering a dormant state. Under these conditions, the phytopathogen spreading process resembles the percolation problem of nearest-neighbor square lattices. The connectivity of the plantation is influenced by the interaction between plants and phytopathogens, which is affected by the susceptibility of the plants. This measures the likelihood of a plant becoming ill after exposure to the phytopathogen. In this way, the propagation process only occurs on susceptible plants, while the resistant ones play the role of physical barriers, which may hinder the formation of large clusters. Under these conditions, the critical susceptibility for monoculture plantation matches the percolation threshold of nearest neighbor square lattices ($\chi_{c}$ = 0.59274621(13)).

We proposed three planting configurations compounded by two plant varieties, namely, well-mixed, intercropping alternating columns, and intercropping alternating diagonals, as agroecological strategies to reduce the spreading of the zoospores. Additionally, we considered the percentage of inoculated cells at the beginning of the propagation process, which can modify the vicinity definition by connecting sites far away from the nearest neighborhood under particular conditions.

We studied the connectivity of the planting configurations by estimating the critical curves in the $\chi_{A}-\chi_{B}$ space. These results are reported as phase diagrams in Figure 6. This can be understood in the following way. For a particular pair of plants with susceptibilities $\chi_{A}^{\prime }$ and $\chi_{B}^{\prime }$, the disease will cover a significant portion of the plantation if the point $\left(\chi_{A}^{\prime },\chi_{B}^{\prime }\right)$ lies above the critical curve, substantially reducing the total yield production. On the contrary, if $\left(\chi_{A}^{\prime },\chi_{B}^{\prime }\right)$ is below the critical curve, the disease will be controlled. We also included information on experimentally determined susceptibilities in the phase diagrams to illustrate the applicability of our results. In all cases, the best planting configuration is the intercropping plantation with alternating diagonals because it has the largest region for the nonpercolation phase. In particular, the serrano–poblano (red triangle in Figure 6) chili pair exposed to the *P. Capsici* strain PcV51 can only be sown in this configuration to avoid the zoospores’ dissemination (see Figure 6) for *I* = 0.00 and 0.01. However, other strategies are required if the percentage of the value of the initial inoculated cell increases (see Figure 6). Another case of interest is the árbol–poblano (red square in Figure 6) pair exposed to the *P. Capsici* strain PcV51. Note that this point is below the critical curve of the well-mixed at Mix = 0.4 and intercropping plantation with alternating columns. In this case, it could be better to use the well-mixed plantation because it allows for sowing more cells with B-type plants, which would be beneficial if the farmer had an interest in harvesting the B-type plants. We recall that we analyzed only fully sown plantations. Therefore, planting density can be considered as another parameter in the model, suggesting the potential for empty cells that could be filled with a more resilient third plant variety. Additionally, planting configurations beyond square lattices can be implemented. In any case, our results can be useful for agroecological and nonchemical planting designs that agree with polyculture systems and promote the sustainable use of soils.

## Figures and Tables

**Table 1:** Experimental determination of the susceptibilities for different *Capsicum* varieties, “chile serrano” ($\chi_{s}$), “chile de árbol” ($\chi_{a}$), and “chile poblano” ($\chi_{p}$), exposed to several *Phytophthora capsici* strains [27].

Oomycete	$\chi_{s}$	$\chi_{a}$	$\chi_{p}$
PcV01	0.60	1.0	0.89
PcV51	0.46	0.27	0.76
PcV77	0.64	0.36	0.04
PcV90	0.40	0.10	0.19
Blank test	0	0	0

## Data Availability

The data that support the findings of this study are available from corresponding authors upon reasonable request.
